# Does Functionality Condition the Population Structure and Genetic Diversity of Endangered Dog Breeds under Island Territorial Isolation?

**DOI:** 10.3390/ani10101893

**Published:** 2020-10-16

**Authors:** José Manuel Alanzor Puente, Águeda Laura Pons Barro, Manuel Rafael de la Haba Giraldo, Juan Vicente Delgado Bermejo, Francisco Javier Navas González

**Affiliations:** 1 Serveis de Millora Agrària i Pesquera (SEMILLA), Producció Agrària del Àrea Tècnica Àgraria, Conselleria d’Agricultura, Pesca i Alimentació, Majorca, Govern Illes Balears, 07009 Palma, Spain; jalanzor@semilla-caib.es (J.M.A.P.); apons@semilla-caib.es (Á.L.P.B.); 2 Department of Genetics, Faculty of Veterinary Sciences, University of Córdoba, 14071 Córdoba, Spain; ge1hagim@uco.es (M.R.d.l.H.G.); juanviagr218@gmail.com (J.V.D.B.)

**Keywords:** ratting and hunting, guard and shepherding, functionality, conservation strategies, pedigree-based assessment, population structure

## Abstract

**Simple Summary:**

Early references to Ca de Rater and Ca de Bestiar endangered autochthonous breeds were witnesses to their exceptional ratting/pet and shepherding/guard skills for centuries. Studbooks or associations promote an increase in the number of effectives and their genealogies. Genetic diversity parameters were evaluated along the history of definition of both dog breeds. Guard or hunting purposes condition an increased registration of genealogical information. Hunting animals have more complete genealogies and need more time to select breeding animals. Male guard dogs are preferred over females due to their suitability for guarding. Selection for performance acts as a diversity promoter and breeding policy driver. The uses or purposes for which certain breeds were selected condition the genetic diversity evolution of endangered breeds, even if these share the same geographic isolation conditions.

**Abstract:**

Despite the undefinition of the origins of Ca de Rater (CR) and Ca de Bestiar (CB) dogs, references to these endangered autochthonous breeds highlighted their ratting/pet and shepherding/guard skills for centuries. Genealogical historical records were traced back to founders. Founder number in the reference population (146 and 53 for CR and CB, respectively), historical and reference maximum generations traced (eight and seven for CR and CB, respectively), and historical average number of complete generations (1.04 for both breeds) were determined. Structure assessment revealed the existence of subpopulations regarding criteria such as breeders (75 and 17), breeder location (32 and eight), owners (368 and 198), and owner location (73 and 51) for CR and CB, respectively. Average inbreeding (F) within breed subpopulations ranged from 0.27–1.20% for CB breeders and the rest of subpopulation criteria for both breeds, respectively, except for CB owners and owner location. F ranged from 0.27–1.41% for CB historical population and CR current population, respectively. The study of genetic diversity revealed a relatively similar genetic background between subpopulations. Average coancestry between and within breeds suggested a similar evolutionary process. However, Mann–Whitney U test determined significant differences for diversity parameters (F, ΔR, coancestry, nonrandom mating degree, maximum, complete, and equivalent generations, ΔF, and genetic conservation index) between breeds and their functionalities. Conclusively, functionality in dog breeds may determine the genetic diversity evolution of endangered breeds, even when these share the same geographic isolation conditions.

## 1. Introduction

An analysis of the historical records of the Majorcan Ca de Rater (CR) suggests rather undefined origins [1]. The first references of the CR breed were described by the Archduke Lluís Salvador of Austria [2] around 150 years ago. Contextually, the study of its relationships with other canine populations supports a presumable ancient origin [3] linked to the Egyptian trunk, which derived in a fully functional ratting dog [4]. This labor not only provided the breed with its name—CR or Ratter dog—but also accompanied and defined the connection of the breed with humans until now [4].

The breed definition may have parallelly occurred with the Majorcan repopulations of Valencian towns such as Taberna (Gallinera valley) in the 17th century, who potentially introduced a Majorcan type of dog which fitted the morphology and purposes of a rat terrier [3]. Reciprocally, other authors ascribe a more recent origin of the breed, to the arrival of Valencians in the Albufera (lagoon) of Majorca to grow rice (to the town of Alcudia and its extension to the towns of Muro and Sa Pobla) at the beginning of the 20th century. These new islanders [5] may be responsible for the introduction and cross of their potentially morphologically and functionally affine animals with the individuals of autochthonous Majorcan breeds [4], to exterminate the abundant rats of the area and their detrimental effects on health and crops [1]. In line with this testimony, certain references named the breed as Majorcan fox terrier, potentially suggesting its relationship with an English namesake [1]. 

Gradually, CR spread across many other Spanish regions [4]. The influences received through the process of conformation and definition of the current population made this dog an exceptional companion animal. However, the main function for which CRs were bred and selected was its greater ability to hunt rats and other nonhunting species [3]. Additionally, the use of CRs in rabbit hunting started to be developed as an attempt to take advantage of their natural instinct toward capturing small mammals or birds. The CR is used to search dirty and river banks where other larger-size hunting breeds cannot access for rabbits, making using of its improved olfactory, sight, and hearing aptitudes, resulting extremely useful in its application for hare coursing, partridge pointing, or thrush retrieving. The breed’s reconsideration may be linked to the revalorization of sustainable traditional hunting techniques, agricultural and natural area maintenance, and culture conservation. 

Ca de Bestiar (CB) origins are undefined [1]. The first morphological descriptions of the breed date from the 19th century [4]. The oldest references suggest the breed could descend from the so-called Alano or shepherd dog, highlighting its contribution and helpful role to the conquer of Majorca by Jaime I of Aragon [1]. Another hypothesis [4] establishes the origins of the CB as being linked to mastiff dog standards, as described by Archduke Lluís Salvador of Austria [2]. After these mastiffs were introduced to Majorca and crossbred with local breeds, CB reduced its size and its character became relatively less complex, making it suitable for the custody of autochthonous cattle and as a house guardian against unwelcome visitors [4]. CB’s current and most relevant application as a shepherd dog to drive all kinds of livestock (sheep, goats, pigs, cows, or even turkeys) conjoins with its role in defending houses against strangers (potentially stemming from its former utilization as a fighting dog). Shepherds used to spend almost all their lives alone in the mountains and needed protection against other dogs and thieves; thus, testing dogs’ ability to protect them was a common practice. 

Despite the common traditional and popular recognition of both breeds, it would not be until the 1970s when the recovery and orderly breeding of these populations began. During the 1980s, the Board of Native breeds of Majorca implemented programs to promote breed recovery. The Club of Ca de Bestiar was unofficially established in 1978, although the breed standard was not drafted until 1980. The standard established the characteristics and was used as a valid reference for determining both the morphological and the phaneroptical adscription of animals to the population and to provide advice on reproduction, which laid the basis for the breed’s recovery. The official recognition of the Club of Ca de Bestiar did not occur until 2001, when Royal Decree 558/2001 was published. On the other hand, the Spanish Club of Ca de Rater Mallorquí was set in 1990 but was not officially constituted until 2001 (Royal Decree 558/2001). The standard of the breed was officially recognized in 2004 (APA Order/807/2004). Since then, both breeds have been exposed to a high risk of loss of genetic diversity resulting from a long process of breeding during which foreign breeds contributed to the loss of their genetic identity from the 1950s [3]. This is a common framework, since, during their definition process, many breeds are characterized by reduced genetic diversity related to a small number of founders. This situation promotes the overrepresentation of the allelic pool of population founders in later generations, as these animals often lay the basis for the breed standard.

Initial stages of breed morphofunctional standardization may promote the cross between close relatives to find and fix the characteristics or qualities that define a breed. This initial mass selection is carried out without considering the possible harmful effects derived from the loss of simultaneous genetic diversity and the increase in other parameters such as inbreeding. Concerns on the potential effects of inbreeding and reduced diversity on health, functionality, and welfare in dog breeds have led to a call for improved genetic management practices [6]. Functionality or the purpose to which breeds are aimed conditions the patterns that drive genetic diversity mechanisms. However, authors such as Pedersen et al. [7] claim that the importance of functionality as a genetic diversity driving agent may have become masked by other more popular factors such as conformation or morphology.

Dog breeds have traditionally been classified depending on their use, which tended to result in some genetically unrelated breeds being grouped together, particularly when dogs of widely different geographical origin were considered [8]. As a result, evaluating the function for which breeds are currently used may virtually enable tracing back modern dog breeds to their origins [7]. Breeds traditionally linked to a certain functional purpose may have evolved in the context of the original use on which the human–animal relationship was built (whether it was ratting, pointing, retrieving, coursing, guarding, shepherding, racing, the hunting of unique types of game, or activities as obscure as bull-baiting or even dog-fighting). The evolution of human necessities changed owner and breeder priorities in terms of which qualities should be considered when selecting animals for their function. This becomes especially patent in equines [9,10] and dogs [7] for which functionality may have played a pivotal role. Breeding practices for conformation replaced better performance criteria due to the displacement of several work breeds to assume a pet role. 

When selecting for an enhanced performance, breeding practices may less likely lead to pronounced changes in basic form and function and, hence, in the underlying diversity. Contrastingly, when selecting for conformation, breeding practices may lead to significant changes in the appearance and performance skills of many breeds, sometimes distorted to extremes [7]. Although, this situation may have affected dog breeds through their uses worldwide, certain factors may have acted as mitigators or promoters of genetic diversity processes. In this regard, the contextual situation of dog breeds, their worldwide expansion, or their popularity may determine the conservation patterns to implement. Conditioning factors of genetic diversity, such as functionality, can be decisive in guaranteeing the future of breeds. Detailed genealogical information of endangered populations, their genetic diversity, the evaluation of their population structure, and conservation practices or breeding policies implemented have become indispensable tools for the development of conservation programs, as the value of reproductive individuals is determined considering their ancestry, which strictly confers an economic basis to inbreeding control and mating management.

For these reasons, the objectives of this work were as follows: (1) to study the evaluation of the integrity of the historical pedigree record of CR and CB, controlling the repercussions of the ancestors and founders; (2) to evaluate the current population structure, genetic variability, and the connections between genetic and demographic parameters, measuring the existing genetic flow and quantifying the risk of loss of genetic diversity, evaluating the degree of threat to which each breed is exposed to suggest effective conservation and selection strategies; (3) to analyze whether functional criteria and idiosyncrasies related to functional breeding may have conditioned genetic diversity and population structure throughout the process of isolation and definition of each breed, even when they shared a common territorial niche. This model can act as a tool to assess the degree of endangerment to which dog breeds may be exposed, which may help implementing effective conservation strategies and selection practices that could be extensible to other populations of dogs and other endangered small populations.

## 2. Materials and Methods

### 2.1. Animal Records and Software

The pedigree records used in this study were provided by the Ca de Rater Mallorquí Club (for CR) and by the Associació de Criadors i Propietaris del Ca Pastor Mallorqui—Club del Ca de Bestiar (for CB). The complete pedigree file includes 1810 animals (46.91% sires; 53.09% dams) born between January 1987 and September 2017 for the historical population of CR. Contrastingly, for CB, the historical pedigree file includes 385 animals (49.61% sires; 50.39% dams) born between June 1994 and April 2018. Genealogical information of each dog was traced back to its ancestors and analyzed. Molecular breed assignment analyses were performed to check for breed assignment percentage (breed purity) and to parallelly detect the potential introduction of other breeds to ensure the reliability of our results using the International Society of Animal Genetics (ISAG) Dog core short tandem repeat (STR) panel with 22 markers (Table S1, Supplementary Materials). The Canine ISAG STR Parentage Kit (2014) is an optimized reagent kit for the analysis of the 22 short tandem repeat (STR) loci recommended by the International Society of Animal Genetics (ISAG) in 2014 for canine parentage determination, allowing us to check the validity of the pedigree file. Amplification and genotyping protocols could be consulted in appliedbiosystems [11]. Population structure and genetic diversity evaluation was performed on the historical pedigree information referred to above and on the currently living populations of both breeds (1468 animals—48.23% sires and 51.77% dams—born between May 2001 and September 2017 for CR; 307 animals—50.16% sires and 49.84% dams—born between April 2005 and April 2018 for CB). 

Demographic and genetic diversity parameters were evaluated using ENDOG (v4.8) software [12] and CFC software [13] on all datasets.

### 2.2. Genealogical Information Analysis

The maximum number of offspring per sires and dams and mean offspring number per male or female were computed by evaluating the trends described by birth number during the period that the study comprised. Pedigree completeness index (PCI) was assessed through the maximum, complete, and equivalent number of generations traced [14]. The study of the genealogical information was computed by calculating the percentage of known individuals from the first to the fifth generation (from parents to great-great-grandparents). 

### 2.3. Analysis of Breeding Policies

Generation length [15] and the average age of parents at the birth of their offspring (used for reproduction or not) were calculated for each of the four gametic pathways: sire to son, sire to daughter, dam to son, and dam to daughter. 

### 2.4. Genetic Diversity

Genetic diversity was evaluated through the parameters described below.

#### 2.4.1. Identity by Descent (IBD) Genealogical Estimators

**Figure 1:**
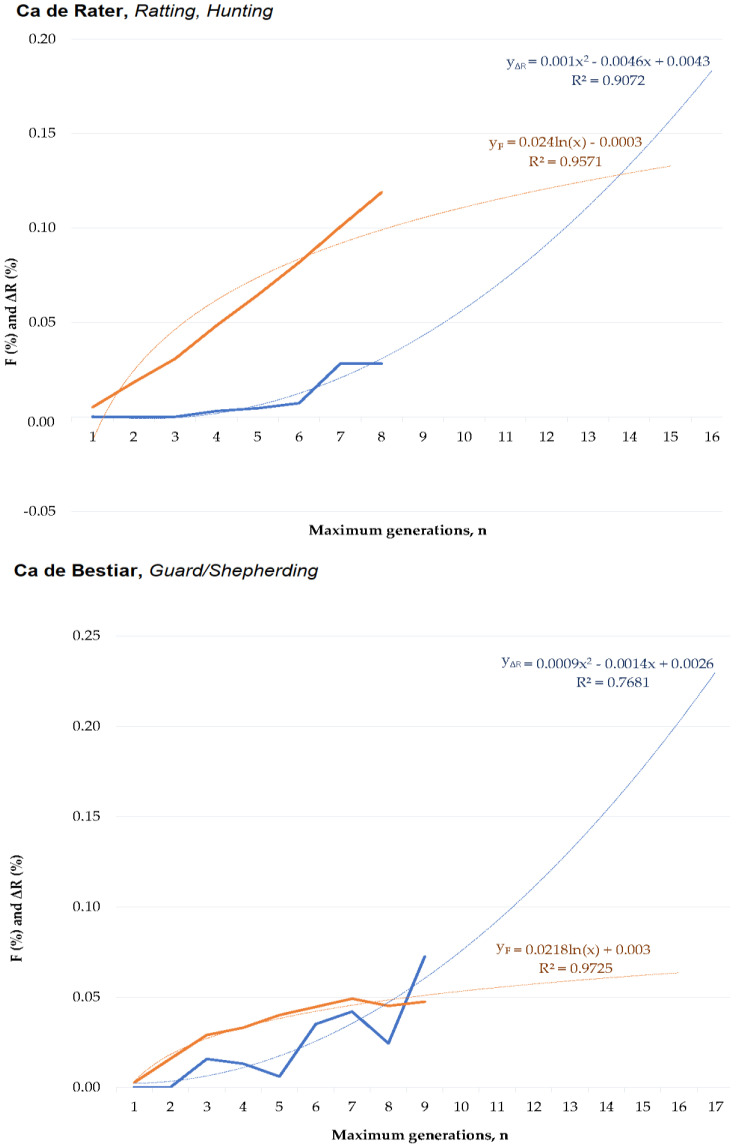
Logarithmic and quadratic regression equations for mean inbreeding (F) and relatedness coefficients (ΔR) from the first to fifth generation and predicted inbreeding (F) and relatedness coefficients (ΔR) from the sixth to 15th generation in the Ca de Rater (CR) and Ca de Bestiar (CB) dog breeds.

**Figure 2:**
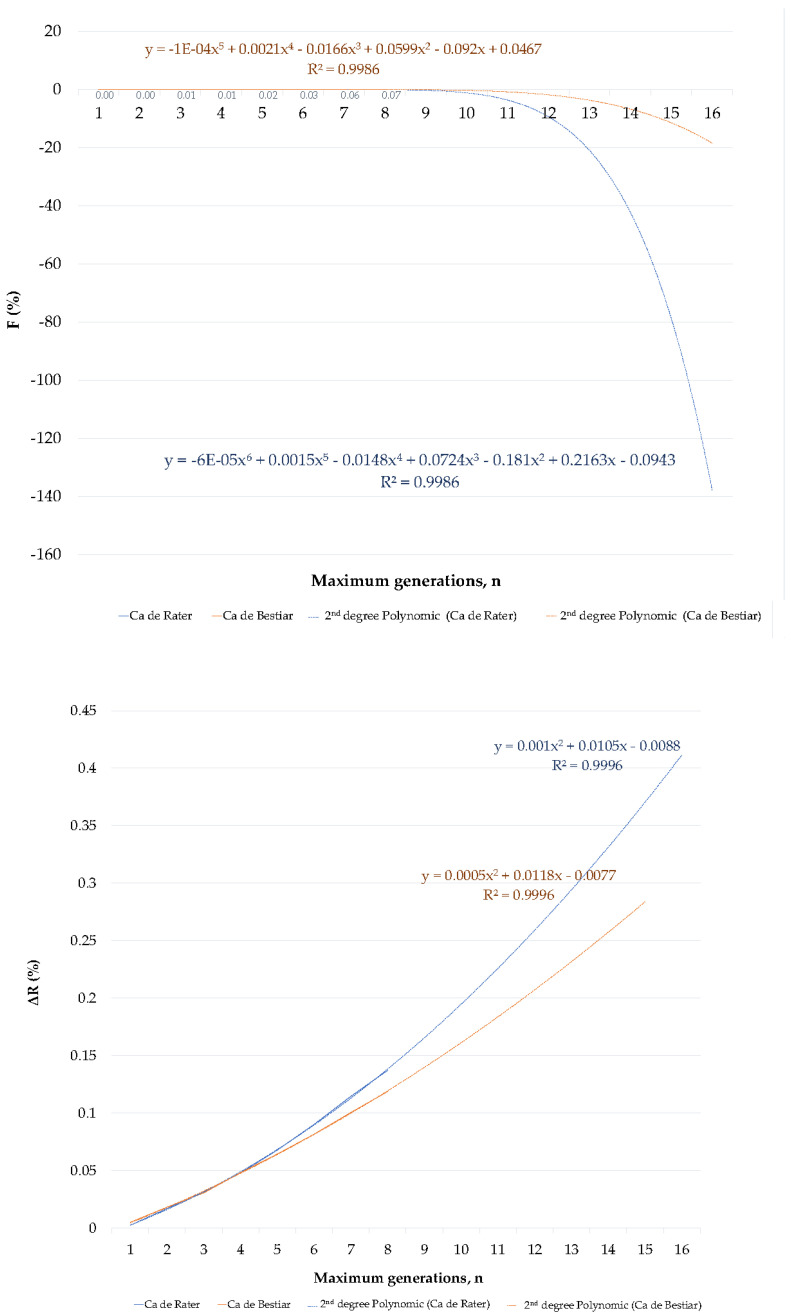
Comparative analysis of quadratic (second-degree polynomic) regression equations for mean inbreeding (F) and relatedness coefficients (ΔR) from the first to fifth generation and predicted inbreeding (F) and relatedness coefficients (ΔR) from the sixth to 15th generation in the Ca de Rater and Ca de Bestiar dog breeds.

Individual inbreeding coefficient (F) was computed as described by Luo [16], the average relatedness (ΔR) of each individual was computed as described by Gutiérrez and Goyache [17], and coancestry (C) coefficient was computed as described by Leroy et al. [18]. The individual rate of inbreeding ($\bar{\Delta F}$) for the generation was computed as suggested by Gutiérrez et al. [19]. Mean ΔR and F per generation were used to issue linear and quadratic regression equations to describe and predict the evolution of both coefficients up to 15 generations, as suggested by Navas et al. [20]. Regression equations, their graphical depiction, and their comparison between functionalities are shown in Figure 1 and Figure 2. 

The individual rate of coancestry ($\bar{\Delta C}$) for the generation was computed as suggested by Cervantes et al. [21]. Assortative mating rate or nonrandom mating degree [22] was assessed to determine the deviation of mating rates from Hardy–Weinberg proportions through its relationship with inbreeding coefficients as suggested by Wright [23]. GCI (genetic conservation index) was computed according to the descriptions in Oliveira et al. [24].

#### 2.4.2. Founder Analysis

The effective number of founders (*f_e_*) was computed as described by Lacy [25], while the effective number of ancestors (*f_a_*) necessary to explain the complete genetic diversity was computed as described by Boichard et al. [26] to account for the genetic variability losses caused by population bottlenecks [27]. The effective number of founder genomes (*f_g_*) was computed as defined by Lacy [25] as the inverse of twice the population individuals’ average coancestry [28]. The expected marginal contribution of each major ancestor *j* was computed as its expected genetic contribution independent from the contributions of the other ancestors [26]. The contributions to inbreeding of nodal common ancestors (with the largest marginal genetic contributions) were computed according to Colleau and Sargolzaei [29].

The mean effective population size ($\bar{N_{e}}$) was computed as described by Wright [23] as the size of an idealized population which would give rise to the rate of inbreeding, or the variance change rate in gene frequencies observed in the population. The number of equivalent subpopulations was computed as described by Cervantes et al. [30]. Genetic diversity (*GD*) was computed as described in Lacy [25]. GD lost in the population since the founder generation was estimated by deducting GD from 1. Unequal founder contributions to GD loss were computed as described by Caballero and Toro [28]. The difference between *GD* and GD * indicates the *GD* loss accumulated since the population foundation [31]. Finally, the effective number of non-founders (*Nef*) was computed following the premises in Caballero and Toro [28] to describe the relationship between the effective number of founders and founder genome equivalents.

#### 2.4.3. Owner and Breeder Pack Relationships

Nei’s minimum genetic distance [32] among breeders, breeders locations, owners, and owner locations were computed to assess interherd relationships. In our case, we evaluated the existence of potential lines when breeders and owners and their locations were considered as the segregation criteria. Dendrograms for owners and breeders for both breeds were constructed using the construct Unweighted Pair-Group Method using Arithmetic averages (UPGMA) Tree task from the Phylogeny procedure of MEGA X 10.0.5. [33]. 

### 2.5. Functionality Impact on Demographic and Diversity Parameters

To evaluate the impact of functionality on demographic and diversity parameters, historical and current populations were subdivided into two groups separately considering each of the breeds and their functionality (CR, ratting/hunting; CB, guard/shepherding). 

The Shapiro–Francia W’ test (for 5 ≤ *n* ≤ 1000 samples) was performed to study data distribution using the Shapiro–Francia normality routine of the Stata Version 15.0 software. Levene’s test was performed to determine the homogeneity of variance across groups using the explore procedure of the descriptive statistics package in SPSS Statistics, Version 25.0, IBM Corp. [34]. As parametric assumptions for diversity parameters were not met (normality and homoscedasticity, *p* < 0.05), Mann–Whitney U and independent median *t*-tests were performed to detect potential differences in population statistics and diversity parameters between CR and CB breeds, respectively (inbreeding coefficient (F, %), average relatedness coefficient (ΔR), number of maximum generations, number of complete generations, equivalent number of generations, individual increase in mean inbreeding (ΔF, %), and genetic conservation index (GCI)). Mann–Whitney U and independent median *t*-tests were performed using the independent samples procedure of the nonparametric tests task of SPSS Statistics for Windows, Version 25.0, IBM Corp. [34]. 

According to Gibbons and Chakraborti [35], the estimated probability of a Type I error was controlled (in the sense of being reasonably close to the attainable level) by the Mann–Whitney test, Student’s *t*-test, and independent median *t*-tests when the variances are equal, regardless of the sample sizes. However, it was controlled by the alternate *t*-test (independent median *t*-tests) for unequal variances with unequal sample sizes.

### 2.6. Publication Ethics Statement

Ca de Rater Mallorquí Club and Associació de Criadors i Propietaris del Ca Pastor Mallorqui—Club del Ca de Bestiar gave their informed consent for the use of pedigree data before the study was performed. As biological samples were not taken, further permission was not necessary. The study was conducted in accordance with the Declaration of Helsinki. The Spanish Ministry of Economy and Competitivity through Royal Decree Law 53/2013 and its credited entity, the Ethics Committee of Animal Experimentation from the University of Córdoba, permitted the application of the protocols presented in this study as cited in the fifth section of its second article, as the animals assessed were used for credited zootechnical use. This national Decree follows European Union Directive 2010/63/UE, from 22 September 2010.

## 3. Results

### 3.1. Genealogical Information Analysis

Two historical birth peaks occurred for both breeds: from 2002 to 2003 and from 2007 to 2008 in CR; in 2007 and in 2009 in CB. However, a drastic reduction in the number of births of the CR breed was observed from 2008 onward. Such a drastic reduction was not observed for the CB breed. The average historical number of births was 58 and 16 and the years for which the highest number of births were registered were 2007, and 2007 and 2009 (163 and 37 births) for CR and CB, respectively. However, after 2011 there was a progressive decrease in the number of births in the CR breed which never reported the minimum levels reached during the 1987–1997 period. Contrastingly, despite CB presenting remarkable fluctuations in the number of births across years, the highest values found for 2007 and 2009 (37) were never reached again in the history of the breed. For CB, the trends were maintained in time and the values never fell below those for the period from 1994–2001. The historical number of complete generations in the last decade was 1.04 ± 0.79 and 1.04 ± 0.62, for CR and CB, respectively. The historical number of equivalent generations was 1.50 ± 1.09 and 0.89 ± 0.99 for CR and CB, respectively. The completeness index of the pedigree experienced a mean increase per generation of 3.646% and 1.558% when the historical and current populations for CR and CB were compared, respectively. The minimum index of completeness of the pedigree was reached for the fifth generation (percentage of great-great-grandparents known) of the historical population for both breeds, with CR reporting a 1% higher value than CB. In contrast, the maximum completeness index was reached for the first generation (known parents) for both breeds with CR reporting almost 34% higher values than CB. The summary of the results for pedigree completeness index-related parameters between the historical and current populations for both breeds are reported in Table 1. The historical maximum progeny per male was 117 and that per female was 39 in CR, while the same parameters reached values of 18 and 16, respectively, for CB. These numbers reduced to 72 and 33 per male and female, respectively, in the current population of the CR, while historical values remained constant for the current population of CB. The average progenies per male were 1.66 and 1.13 in the CR historical population and reference population, respectively. These values were reduced to 1.00 and 0.81 in the CB historical and reference populations, respectively. Contrastingly, the average progenies per female were 1.45 and 1.00 in the CR historical population and reference population, respectively. These values were reduced to 0.96 and 0.67 in the CB historical and reference populations, respectively. The proportion of females per male was 1.13/1 and 1.05/1 and 1.02/1 and 0.99/1, for historical and current populations in CR and CB, respectively. The progeny of males selected for breeding was around 40–45% in historical and current populations of CB, with the exception of progeny of males selected for breeding in the current population, which was reduced to half this value (21.54%) in the historical and reference populations. Slightly lower values were found for the CR breed, which were around 15% to 18% for the progeny of males and females selected for breeding in the historical and current populations. Progeny analysis results for all studied population subdivisions are presented in Table 1.

The average age of the males and females in reproduction was very similar (~14 years) for the historical and reference populations of both breeds. Generation length was 3.94 and 3.93 years and 3.09 and 4.04 for the historical and reference populations of CR and CB, respectively. The lowest values reported for generation interval were 3.76, 3.63, and 2.70 for the gametic routes of sire to son and dam to son in the historical populations and sire to daughter in the current populations of the CR and CB, respectively (Table S2, Supplementary Materials). Table S2 suggests that the mean age (years) of the parents at the birth of their offspring for the four gametic routes in both breeds was around 4. A summary of the demographic and offspring statistics derived from the analysis of the pedigree of the two breeds is reported in Table 1. 

### 3.2. Genetic Diversity

#### 3.2.1. Identity by Descent (IBD) Genealogical Estimators

Inbreeding coefficients for the historical and current populations were 1.15% and 1.41% and 0.27% and 0.34% for CR and CB, respectively. Despite these relatively low coefficients, highly inbred animals were recorded in the historical and current populations. The maximum percentage of inbreeding (26.41%) was reported for the historical and current populations of CR. CB reached half the value reported for CR in both populations (13.38%). The percentage of inbred animals was 1–2-fold higher in the historical and current populations of CR compared to CB (Table 2). Nonrandom mating rate was 0.00 and −0.01 for the historical and reference populations of CR and CB, respectively, as shown in Table 2.

The average coancestry in the historical and reference populations was 1.24% and 1.41%, respectively, for CR, while it was 0.86% for the historical and reference populations of CB.

#### 3.2.2. Founder Analysis

The results for the analysis of probabilities of gene origin, ancestral contributions, and the loss of genetic diversity are shown in Table 3. GCI reported values around 3 for historical and current populations of CR and values of 2 for historical and current populations of CB (Table 2).

Genetic diversity was around 99% in both breeds. The lowest value was reported for CR, although the differences with CB were not remarkable (98.76%), which was the population for which genetic diversity loss was consequently slightly greater. The loss of genetic diversity due to genetic drift was 0.5% and 0.3% in CR and CB populations, respectively. A value of 1.24% and 0.9% was reported for the genetic diversity loss that could be attributed to bottlenecks and genetic drift for the CR and CB reference populations (Table 3).

The average relatedness (kinship) coefficient was 24.09% and 1.73% in the CR and CB reference populations. For the CR, considering the marginal genetic contributions of ancestors, a single ancestor (identification number 138) explained 9.25% to 14.13% of the genetic pool of the historical population and 8.97% to 13.55% of that of the current population. Contrastingly, for CB, marginal genetic contributions of a single ancestor (identification number 202) explained 9.77% to 15.89% of the genetic pool of the historical population and 4.42% to 8.37% of that of the current population. Additionally, 15 and 31 individuals explained 50% of the gene pool of the current and historical populations of CR and CB, respectively.

Results for effective sizes calculated through the individual inbreeding rate and individual coancestry rate are reported in Table 4. Effective population size calculated through the individual inbreeding rate was 54.35 and 384.62 in the CR and CB historical populations. Comparatively, the effective sizes of the population calculated through the individual coancestry rate of the CR and CB historical populations were 20.08 and 28.90, respectively.

#### 3.2.3. Owner and Breeder Pack Relationships

A total of 67,161 and 19,306 Nei’s genetic distances were considered when the stratification criterion was the owner for CR and CB, respectively. The average Nei’s genetic distance was 0.103 and 0.274 for CR and CB owners, respectively. Contrastingly, for breeder, breeder location, and owner location this parameter was 0.074 and 0.038, 0.021 and 0.042, and 0.025 and 0.072 for CR and CB, respectively. The number of equivalent subpopulations for all population sets was 0.370 and 0.075 for CR and CB, respectively (Table 4). The average numbers of CRs per breeder, breeder location, owner, and owner location were 24.133, 56.563, 4.919, and 24.795, respectively, while the average numbers of CBs per breeder, breeder location, owner, and owner location were 22.647, 12.031, 1.944, and 7.549, respectively. The mean coancestry within the subpopulations for CR breeder, breeder location, owner, and owner location was 0.086, 0.050, 0.116, and 0.034, respectively. For CBs, the mean coancestry within the subpopulations for breeder, breeder location, owner, and owner location was 0.051, 0.034, 0.283, and 0.080, respectively (Table 5). 

Mean coancestry in the metapopulation and autocoancestry reported values of 0.013 and 0.506 for all population subdivisions (breeder, breeder location, owner, and owner location) for CR, while these values were 0.009 and 0.501, respectively, for all population subdivisions (breeder, breeder location, owner, and owner location) for CB. The analysis of population structure through Wright’s F statistics (Table 5) reported that the inbreeding coefficient of a certain individual with respect to the total population (F_IT_) was −0.001 and −0.006 for all subpopulations and criteria considered. The coefficient of inbreeding of an individual with respect to the subpopulation (F_IS_) varied from −0.390 for the subpopulations of the CB breed when the owner criterion was followed to a maximum of −0.023 for the subpopulations of CR when the owner location criterion was considered (Table 5). The correlation between random gametes drawn from the subpopulation relative to total population (F_ST_), i.e., the effect of the subpopulations compared to the total population, reached a maximum value of 0.276 for the owner subdivision or stratification criterion in CB and a minimum of 0.021 for the owner location subdivision or stratification criterion in CR.

The analysis of the structure of owners and breeders revealed that none of them could be considered as the nucleus of the population. We found that 100% of owners used foreign sires in both breeds, and none of them could be considered totally isolated. In total, 173 owners used the maximum percentage of own parents (66.67%) for CB, while 315 owners used the maximum percentage of own parents (25.00%) for CR.

**Figure 3:**
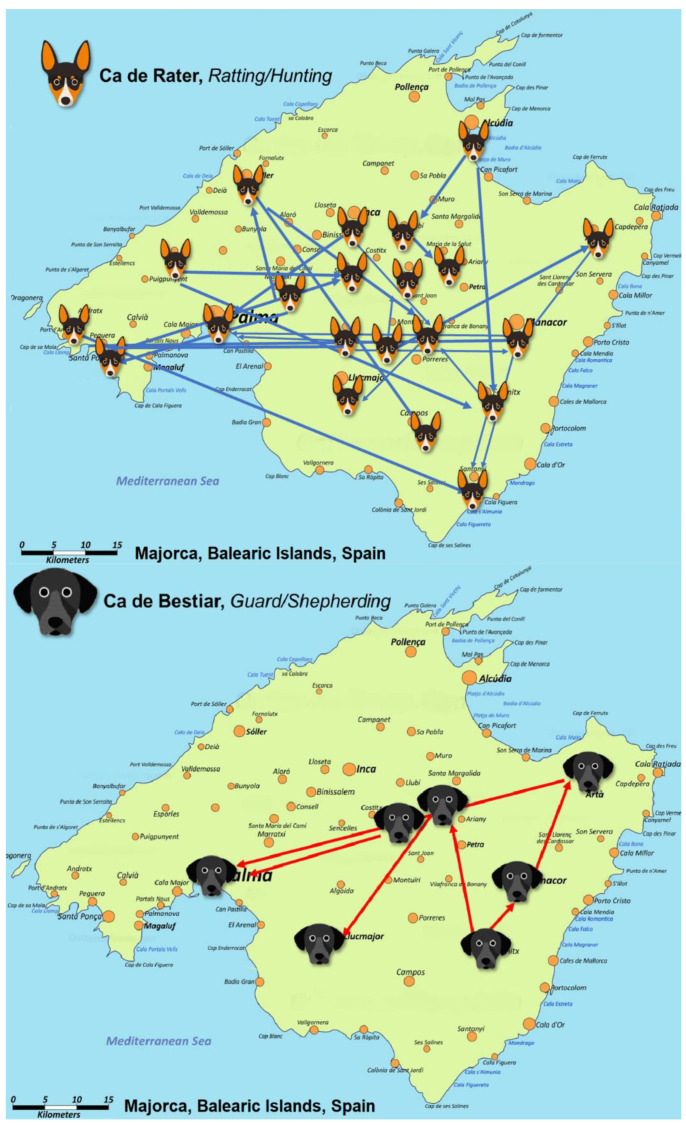
Breeder location connection maps for Ca de Rater and Ca de Bestiar breeds.

In total, 29 pairs of owners of CRs presented the longest genetic distance, which was 0.547. On the other hand, 236 pairs of owners of CBs were distanced by the longest genetic distance (0.508). Figures S1 and S2 display four dendrograms representing all the relationships between populations considering the criteria of owner and breeder for both CR and CB breeds. Breeder dendrograms match the patterns of the initial part of each owner dendrogram, as they constitute the basis from which the population of each breed was historically derived. Figure 3 represents the connection between breeder territorial areas. In the case of CR, two main currents could be observed. The first went from the north of Majorca to the southwest, while the second horizontally crossed the island, forming a bidirectional flux from east to west and vice versa. For CB (Figure 3), an ascending current was formed from the southeastern region of the island upward. This flux of animals would later cross the island diagonally, ending in two locations in the southwestern territories of Majorca, thereby depicting a remarkably more disperse distribution than that shown by CR breeder structure.

### 3.3. Functionality Impact on Demographic and Diversity Parameters

Table S3 (Supplementary Materials) shows a summary of the descriptive statistics of genetic diversity population parameters for historical and current populations of CR and CB breeds. The outputs derived from Mann–Whitney U and independent median *t*-tests (Table S4, Supplementary Materials) report that all genetic diversity population statistics differed between functionalities.

## 4. Discussion

**Figure 4:**
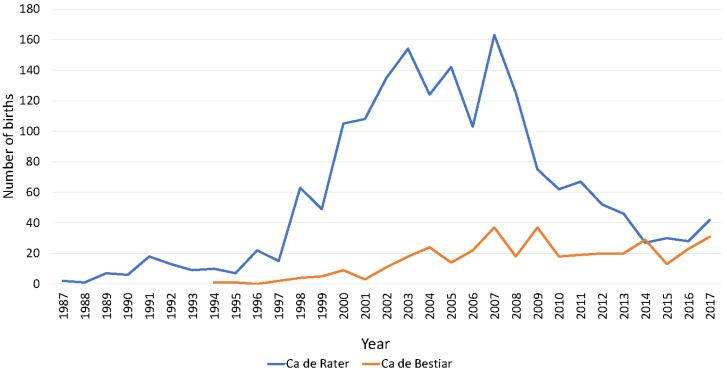
Birth number evolution from 1987 to 2018 for Ca de Rater and Ca de Bestiar dog breeds.

The number of births recorded in the pedigree of CR and CB dog breeds describes an irregular evolutionary tendency (Figure 4). The two peaks in the number of births in CR took place after the official recognition by the Ministry of Agriculture and Fisheries of the Balearic Government that occurred on 28 December 2002 (in 2003 and 2007). This 2007 peak coincides with the first peak of CB, followed by a second peak in 2009. During these years, the activity of these associations was increased by morphological and monographic competitions to publicize their activity and promote the autochthonous breeds of the archipelago, which may explain such an increase.

Pedigree completeness levels were lower than those reported by Leroy et al. [36], who reported common approximate levels for the fifth generation of 100% for internationally recognized breeds (not isolated nor endangered). The lower levels found in the autochthonous breeds in this study may derive from their endangered status and the lack of genetic management occurring in both breeds, as official structures were recognized relatively recently. Contrastingly, our results for PCI (around 60%) may be in line with those of Cecchi et al. [37] for animals belonging to internationally recognized dogs such as Labrador, Golden Retriever, and German Shepherd used as guide dogs. These results suggest the collateral application of certain breeds to purposes that may differ from their consideration as pets, which may condition genealogical information registration from these animals, as, in the latter, other factors, such as conformation, may potentially be rather highly considered.

The low PCI levels found contrasts the levels reported for the common context of dog breeds that are internationally recognized, which may enjoy greater worldwide popularity. Contextually, the low PCI levels for the fifth generation may derive from the fact that genetic management programs of both breeds are still in their first stages of development. The conservation strategies implemented in both breeds started with a low number of effectives on which to support management structures (studbook, association, among others) which are only around 10 to 15 years old, even if a great breeding tradition of these breeds can be found in the archipelago prior to the recognition of the breed [36]. This may be supported by the fact that the mean number of equivalent generations was maintained over time, as suggested by Marin et al. [38].

**Figure 5:**
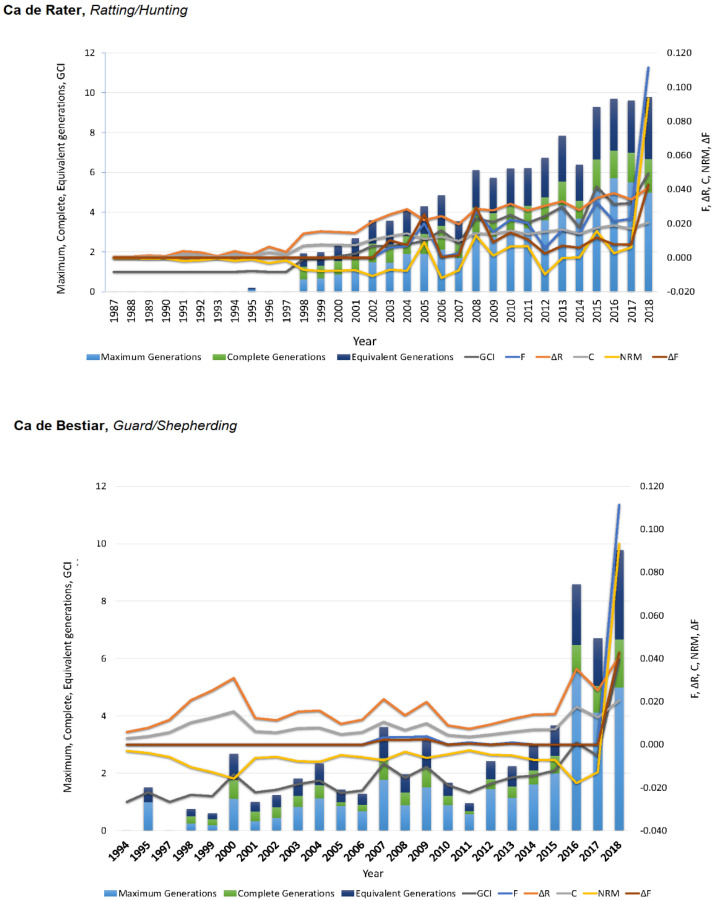
Trends for identity by descend estimators (F, C, ΔF), ΔR, nonrandom mating degree (NRM), and genetic conservation index (GCI) from 1987 to 2018 for Ca de Rater and Ca de Bestiar dog breeds.

Maximum, complete, and equivalent generations in CB were significantly lower than the values reported for CR (Tables S3 and S4, Supplementary Materials; Figure 5). The mean number of equivalent generations (EqG) for both breeds was lower than that observed for Italian Bracco by Cecchi et al. [39], for the Braque Français type Pyrénées by Cecchi et al. [40], and for Ca Mè by Marin et al. [38], with the latter sharing the same territorial isolation conditions as the breeds in our study. The latter breeds differed from those in our study in terms of their hunting application in comparison to the ratting/domestic and shepherding/guard applications of CR and CB, respectively, which may have conditioned the results. In line with our results, the study by Leroy et al. [36] reported similar EqG in breeds of a renowned shepherding/guard background to those reported for the upper limit found for CB. 

Breeder dendrograms lay the historical basis of owner dendrograms; hence, breeder pack structures can be inferred to build the skeleton of the owner pack network (Figures S1 and S2). Both breeds were predominant around the center of the island as opposed to the coastal areas, since it is in these zones where their functional aptitude, linked to production systems (cattle herding and privately owned preserves) and occupational habits of the population (vacation houses), extends.

Generation intervals resembled the values found by Leroy et al. [34] for Basset fauve de Bretagne, French Bulldog, Dogue de Bordeaux, Barbet, Berger des Pyrénées, Beauceron, Epagneul Breton, and Pyrenean Mountain Dog and were somehow lower than those found by Leroy et al. [41] and Cecchi et al. [40] for the breeds recognized by Fédération Cynologique Internationale (FCI). Hunting dogs reported the longest generation intervals, which may be based on the time that breeders take to test the performance of animals before choosing their offspring for the next generation [38]. Guard dogs [36] on average reported similar values of ~5, which were similar to those found in the current population of CB. Shorter generation intervals for French Bulldog and CR may be related to their application as pets, which may make performance testing a secondary or irrelevant position.

In general, mean generation intervals were slightly longer for male offspring (via both sire and dam) than for female offspring, with the highest value being reported for the gametic pathway of sire to son for the current population of CB, which may derive from the higher number of male dogs than female dogs whose age at the birth of their offspring was above the mean. A certain preference for dogs of a particular sex has been reported and may be based on a perceived dimorphic superiority in some desired traits, which may condition the time taken by breeders to determine the validity of a certain animal as a mating animal. For instance, males have normally been reported to be bolder, higher driven, and more aggressive than females, who tend to learn faster, be somehow less territorial, and more biddable. Differences in boldness and shyness are acknowledged predictors of performance in working dogs, with bolder animals being the better performers [42]. Boldness is an individual characteristic providing less aversion to risk or novelty that enables the individual actively seeking out and engaging in social cooperative and competitive interactions [43]. This mirrors the recently reported opinions of working farm dog handlers, who identified boldness in their dogs as a desirable trait. By contrast, although it is possible that male and female dogs may differ in boldness, there is limited evidence of sex differences in the herding style of working farm dogs [44].

The policies of breeders denote the use of certain males or females which may be more popular among the individuals in the population. Contextually, having a low replacement rate could contribute to generation elongation which has been reported for other endangered dog or equine breeds [20]. Both species, equines and canines, may share certain similarities with respect to the over-consideration of the value provided to ancestors and the conditioning effect of such ancestral value on the relevance of the individuals in the current population. 

A slightly higher number of females than males selected for breeding was reported in both breeds. However, female to male ratio inverted in CB, which suggests a certain interest for breeders and owners in the males of the CB breed, which could be presumably linked to their application as guard or shepherding animals. In nature, for males, territorial defense versus access to female success ratio may be balanced by ameliorative reproductive success, whereas females have fewer direct advantages from this ratio; hence, the energy invested in the defense may be detracted by the functions related to the sex-specific behaviors linked to reproduction [43]. The same authors suggested that male dogs tend to be more social and to engage more in dog/human contact than females, whereas, in cooperative behavior in trying to solve a problem, the opposite trend has been found. These features may condition breeder preference when the selection criterion is performance for a specific outcome, such as guarding [43], as already discussed. However, hunters reported no differences between males or females in hunting performance [45], even if a certain interaction between breed sex conditioning effect was suggested, with male Dachshunds and West Highland White Terriers being found to be significantly more trainable than females of the same breeds [46].

Mating between dogs that are related beyond second cousins (F < 0.0156) has been reported in small territorially isolated populations such as the ones in our study as suggested by Marin et al. [38]. However, these values should be cautiously regarded as low levels of pedigree completeness indices for fifth generation may denote that diversity estimators are underrated. For instance, Marin et al. [38] reported higher F values for Ca Mè, which were supported by considerably higher levels of PCI across generations, which may derive from the greater importance of the knowledge of genealogy in hunting dogs when compared to guard dogs or even domestic dogs without a marked functionality. According to Navas et al. [20], remote levels of inbreeding may not determine a relevant impact on health, although they may still give way to a marked increase in homozygosity levels, which is often sought when the breed is defined in accordance with a certain standard, whether it is morphological, functional, or zootechnical.

In our study, inbreeding levels showed an increasing trend which stabilized around 14 or eight maximum generations for CR and CB, respectively (Figure 1). This contrasts ΔR levels which increased over the years, describing a rather exponential curve, which may suggest the progressively increase in breeders using related animals for the obtention of their litters (Figure 2). The values for F and ΔR fall within the range of values reported by the study of Leroy et al. [41] for 61 breeds. These authors suggested that F ranged from 0.2% (for Czechoslovakian Wolfdog) to 8.8% (for the Pyrenean Shepherd). Concretely, CB values were the same as those reported by the Romagna Water Dog, while CR values were the same as those reported by the Italian Mastiff. ΔR levels ranged from 0.4% (for poodle) to 8.8% (for Saint Germain pointing dog), while CB reported the same values as Cairn Terrier and CR reported the same values as Cavalier King Charles Spaniel. These results suggest a weaker ancestral connection in breeds of a remarkable pet application than in those with a rather distinctive functionality such as hunting, shepherding, or guarding. 

All diversity parameters differed highly statistically between guard/shepherding dogs and ratting/hunting/pet dogs (Tables S3 and S4, Supplementary Materials), with the latter reporting double the values for F, ΔR, $\bar{\Delta F}$, and GCI. Pedersen et al. [7] suggested that the diversity parameters of conformation-type breeds differed from that in breeds heavily used for performance, with the latter clustering more closely with village dogs. The same authors concluded that, in comparison to their village dog relatives, all modern breed dogs exhibit reduced genetic diversity, which was even more reduced among breeds under selection for show/conformation, which may suggest that selection for performance may act as a diversity promoter.

Provided the historical territorial isolation to which CR and CB have been exposed, a solid interconnection between available genetic resources through the implementation of appropriate breeding practices may have been attempted, as suggested by nonrandom mating results in CR, which could be said to be in genetic equilibrium. This may suggest that this population will be balanced as soon as no external influence such as selection can promote the increase in mutation rates. The negative values for CB may imply that mating among the animals may not have been performed seeking particular phenotypical, phaneroptic, or functional characteristics. These values contrast the ones obtained for Ca Mè by Marin et al. [38], whose positive value of 0.02 may imply that certain characteristics were sought after along the trajectory of the breed, such as specific desirable coat patterns as a complement of a greater adaptability or suitability of the animals for hunting.

The number of equivalent subpopulations below 2 indicates a high structuration of both populations (0.37 and 0.075 for CR and CB, respectively) whose values were lower than those found for Ca Mè [38]. According to Fernández et al. [47], maintaining subdivided populations has the advantage of a reduced extinction risk derived from potential natural catastrophes or health-related factors, as these events may only affect reduced individual groups. Similarly, the higher levels of genetic diversity may have been attained when a certain population was subdivided in time in as many groups as possible, while considering that subdivision in lines may be detrimental due to the smaller effective size of each subline, which may, thus, translate to an increased level of inbreeding.

The F_ST_ value of zero suggests no population structuring or subdivision, i.e., complete panmixia or random mating. The concept of panmixia is opposed to the concept of assortative or nonrandom mating (Table 2). Panmixia via weak positive assortative mating has been reported to be typical for natural animal populations, while disassortative mating is rare or absent [48], as reported for CB (Table 2). In this context, repeated backcrossing may be considered as a particular application of disassortative mating [49]. Generally, disruptive selection will indirectly favor positive assortative mating to avoid producing less fitted offspring; conversely, stabilizing selection will favor negative assortment [50]. Additionally, for similar reasons, nonrandom mating can evolve in response to inbreeding or outbreeding depression [51].

A value of one for F_ST_ implies that all genetic variation is explained by the population structure, mainly conditioned by the existence of barriers to gene flow (geographical, linguistical, sociocultural, and even economical) and, therefore, that the two populations examined do not share any genetic diversity. At a breed level, F_ST_ values are always expected to be below 0.05, as this may be the lower limit for species differentiation. Nevertheless, computing F_ST_ values can report very important information about the relationships among lower-scale genetic subdivisions of a population, such as breeds or varieties, or those linked to specific features such as coat color or even functionality.

This becomes even more patent when values for F_ST_ are comparatively interpreted with F_IS_ values. At a breed level (F_ST_ below 0.05 context), when owner, breeder, and their locations are considered as criteria of population subdivision, negative values of F_IS_ may address the existence of a certain disequilibrium in breeding policies acting in favor of an unexpected mating rate of unrelated animals under a model of random mating. This imbalance may derive from the fact that breeders and owners may tend to mate animals that have desirable aesthetic qualities or higher performance in different skills as a way to improve the resulting puppies through the complementarity between features or abilities displayed by both parents, which was also supported by the results of the Mann–Whitney U test (Table S4, Supplementary Materials).

In this context, as indirectly suggested by Robertson [52], if mating occurs at random within a line or breed, then the decline in heterozygosity lags one generation behind the genetic drift. This implies the stabilization of a breed population if the mating of distant relatives is performed, or if mating between relatives is avoided as much as possible. The mating together of distant relatives within a line or breed leads to lower initial inbreeding but a higher final rate of approach to the limit, as opposed to when mating of close relatives is promoted. Reduction of genetic drift to a minimum requires the formation of permanent sublines. If sublining is only partial (division into groups whose immediate ancestors may differ but which become identical by descent at some distance back in the pedigree), then the proportional rate of decline in heterozygosity is equal to the rate of approach of genetic variance between lines to its final value. 

Our results were supported by negative F_IS_ values, which may be indicative of individuals in a population subdivision being less related than could be expected under a model of random mating. This could be explained by the restrictive breeding policies expected from dog breeders, which may be reinforced under conditions of territorial isolation such as those in the area of expansion of these breeds.

According to Calboli et al. [53], shepherding breeds may be characterized by a percentage of founders above 6%, with this parameter being reduced to 2–3% in hunting dogs. In the case of the breeds considered in our study, the effective number of founders was in the range of values reported by Leroy et al. [36]. However, values were around three times higher in CB and 0.5 times higher in CR when compared to the values reported by Marin et al. [38] for Ca Mè.

The ratio of f_e_/f_a_ suggests that the genetic information of founders has been preserved through time. In our study, values ranged from 0.30 in CB to 0.55 in CR, which were similar to those values reported for French breeds by Leroy et al. [41]. The study by Voges and Distl [54] showed that bottlenecks found in dog breeds had a higher impact when they presented a lower *f*_e_/*f*_a_ ratio, which accounted for a greater difference between founders and ancestors (ancestors without or with known/partially known genealogy).

These results evidence that founding genotypes are still representative in current populations of CB and CR. The values found for both breeds also suggest that, although bottlenecks may have occurred, they have not had an effect. Bottlenecks need not lead to or save a population of a breed from extinction. A loss of diversity as a function of bottlenecks occurring in populations could manifest itself from the two options described. Thus, simultaneously with this loss of diversity, deleterious mutations could have been both eliminated and fixed in the population, which could have led both to the sanitation of populations and to an increase in the threat of extinction to which breeds are exposed [20].

In this context, according to Broeckx [55], once reproductive aims covering the specific framework of dog breeds have been defined and the problems for that particular populations have been identified, the approach to pursue and reach possible solutions is similar, as it always stems from the identification of un(desirable) phenotypes and the genotype behind them.

On occasions, sublining can derive from the reproductive policies historically followed by breeders as suggested by Marin et al. [56] and Marin et al. [57]. In this regard, mating planification may involve certain animals which stand out for a particular phenotypic or functional feature, although this may not have been the case for CR and CB, as indicated by the degree of nonrandom mating found. For instance, CR and CB homeowners and breeders may have historically developed intra-subpopulation breeding practices that may have alternatively promoted the genealogical disconnection of the animals participating in mating which may promote genetic diversity. These objectives differ depending on each breed’s social context, but they can be achieved following two opposite approaches: selection against undesirable features or promotion of desirable ones.

## 5. Conclusions

The consolidation of genetic management structures such as studbooks or associations promotes an increase in the number of effectives and their genealogical information in endangered breeds. Guard or hunting purposes may condition an increased registration of genealogical information provided the value of ancestors may be considered an evidence of the potentially higher value of individuals. Hunting animals need longer time intervals until animals are selected for breeding, and genealogical information in hunting animals may be more complete than in pet, ratting, or guard/shepherding breeds. Male guard dog generation intervals suggest that they may be preferred over female dogs which may be based on their special suitability to develop guarding tasks. Selection for performance may act as a diversity promoter. Breed functionality and, hence, its social context may condition whether breeding policies focus on the selection against undesirable features or the promotion of desirable ones. Choosing one alternative or the other may depend on whether performance, independently of the task that the dogs are required to develop, is among the selection criteria of the breeds or not.

## Figures and Tables

**Table 1:** Summary of demographic and offspring analysis parameters in Ca de Rater and Ca de Bestiar historical and current population sets.

	Population Set	Ca de Bestiar		Ca de Rater	
Parameter		Historical	Current	Historical	Current
Population size		385	307	1810	1468
Maximum number of traced generations, *n*		7	7	8	8
Pedigree completeness level at 1st generation, (known parents)		48.96	50.33	77.71	84.88
Pedigree completeness level at 2nd generation, (known grandparents)		23.05	25.98	41.56	50.80
Pedigree completeness level at 3rd generation, (known great-grandparents)		10.13	12.22	17.54	17.54
Pedigree completeness level at 4th generation, (known great-great-grandparents)		4.09	5.13	7.45	8.86
Pedigree completeness level at 5th generation, (known great-great-great-grandparents)		1.43	1.79	2.46	2.87
Number of maximum generations (mean ± SD)		1.72 ± 2.22	1.96 ± 2.22	2.35 ± 2.09	2.77 ± 2.09
Number of complete generations (mean ± SD)		1.04 ± 0.62	0.57 ± 0.62	1.04 ± 0.79	1.18 ± 0.79
Number of equivalent generations (mean ± SD)		0.89 ± 0.99	0.97 ± 0.99	1.50 ± 1.09	1.74 ± 1.09
Male %		49.61	50.16	46.91	48.23
Mean number of puppies per male, *n*		1.00	0.81	1.66	1.13
Maximum number of puppies per male, *n*		18	18	117	72
Average age of male in reproduction, years		14.78	14.09	14.30	13.61
Female %		50.39	49.84	53.09	51.77
Mean number of puppies per female, *n*		0.96	0.67	1.45	1.00
Maximum number of puppies per female, *n*		16	16	39	33
Average age of female in reproduction, years		13.16	12.32	14,91	14.07
Female/male ratio		1.02/1	0.99/1	1.13/1	1.05/1
Progeny from male selected for breeding, %		39.13	21.54	14.86	15.29
Progeny from female selected for breeding, %		44.00	40.54	17.74	18.22

**Table 2:** Summary of identity-by-descent estimators, nonrandom mating rate (α), and genetic conservation index (GCI).

	Population Set	Ca de Bestiar		Ca de Rater	
Parameter		Historical (*n* = 385)	Current (*n* = 307)	Historical (*n* = 1810)	Current (*n* = 1468)
Inbreeding (F, %)		0.27	0.34	1.15	1.41
Average individual increase in inbreeding (ΔF, %)		0.13	0.16	0.92	1.13
Maximum coefficient of inbreeding (%)		13.38	13.38	26.41	26.41
Inbred animals (%)		4.68	5.86	13.98	17.17
Highly inbred animals (%)		1.30	1.63	3.70	4.77
Average coancestry (C, %)		0.86	0.86	1.24	1.41
Average relatedness (ΔR, %)		1.73	1.73	2.49	2.83
Nonrandom mating rate (α)		−0.01	−0.01	0.00	0.00
Genetic conservation index (GCI)		2.00	2.10	2.98	3.32

**Table 3:** Summary of the measures of genetic diversity, genetic diversity loss, and analysis of the probabilities of genetic origin.

	Reference	Ca de Bestiar (Both Parents Known) (*n* = 180)	Ca de Rater (Both Parents Known) (*n* = 1367)
Parameter			
Historical population		385	1810
Current population		307	1468
Base population (one or more unknown parents)		205	443
Actual base population (one unknown parent = half-founder)		196.50	403.50
Number of founders, *n*		53	146
Number of ancestors, *n*		61	148
Effective number of non-founders (Nef)		171.99	102.51
Number of founder equivalents (fe)		87.32	66.08
Effective number of ancestors (fa)		26	36
Founder genome equivalents (fg)		57.92	40.18
fa/fe ratio		0.30	0.55
fg/fe ratio		0.66	0.61
Genetic diversity, GD		0.99	0.99
Genetic diversity loss, GDL		0.01	0.01
Genetic diversity in the reference population considered to compute the genetic diversity loss due to the unequal contribution of founders, DG		0.99	0.99
GDL due to bottlenecks and genetic drift since founders (GBDr)		0.01	0.01
GDL due to genetic drift since founders (GDr)		0	0
GDL due to unequal founder contributions		0.01	0.01
Ancestors explaining 25% of the gene pool (*n*)		4	5
Ancestors explaining 50% of the gene pool (*n*)		10	13
Ancestors explaining 75% of the gene pool (*n*)		21	36
Average individual increase in inbreeding (ΔF)		0	0.01
Average relatedness (ΔR)		0.02	0.25

**Table 4:** Statistical results for effective population size calculated on the basis of the individual inbreeding rate, the individual coancestry rate, and the number of equivalent subpopulations.

Parameter	Ca de Bestiar Historical (*n* = 385)	Ca de Rater Historical (*n* = 1810)
Effective population size based on the individual inbreeding rate	384.62	54.35
Effective population size based on the individual coancestry rate	28.90	20.08
Number of equivalent subpopulations	0.075	0.37

**Table 5:** Summary of Wright’s fixation statistics.

Parameter	Ca de Bestiar				Ca de Rater			
	Breeder	Breeder Location	Owner	Owner Location	Breeder	Breeder Location	Owner	Owner Location
F_IS_ (inbreeding coefficient relative to the subpopulation)	−0.051	−0.0323	−0.390	−0.084	−0.082	−0.041	−0.118	−0.023
F_ST_ (correlation between random gametes drawn from the subpopulation relative to the total population)	0.0427	0.0255	0.276	0.072	0.075	0.0383	0.105	0.021
F_IT_ (inbreeding coefficient relative to the total population)	−0.006	−0.006	−0.006	−0.006	−0.001	−0.001	−0.001	−0.001
Mean inbreeding within subpopulations	0.0027	0.0027	0.003	0.003	0.012	0.012	0.012	0.012
Mean number of animals per subpopulation	22.647	12.031	1.944	7.549	24.133	56.563	4.919	24.795
Total Nei’s genetic distance	120	21	19306	1225	2701	465	67161	2556
Average Nei’s genetic distance	0.042	0.025	0.274	0.072	0.074	0.038	0.103	0.021
Mean coancestry within subpopulations	0.051	0.034	0.283	0.080	0.086	0.050	0.116	0.034
Autocoancestry	0.501	0.501	0.501	0.501	0.506	0.506	0.506	0.506
Mean coancestry in the metapopulation	0.009	0.009	0.009	0.009	0.013	0.013	0.013	0.013
Subpopulations	17	8	198	51	75	32	368	73

## Supplementary Materials

The following are available online at https://www.mdpi.com/2076-2615/10/10/1893/s1, Figure S1. Dendrograms constructed from Nei’s genetic distances between owners and breeders in Ca de Bestiar breed; Figure S2. Dendrograms constructed from Nei’s genetic distances between owners and breeders in Ca de Rater breed; Table S1. International Society of Animal Genetics (ISAG) Dog core STR panel with 21 markers showing homozygosity (Ho) and heterozygosity (He) values for each marker [56]; Table S2. Generation intervals (years) and mean age (years) of the parents at the birth of their offspring for the four gametic routes in the Ca de Bestiar and Ca de Rater dog breeds; Table S3. Summary of the descriptive statistics of genetic diversity population parameters for Ca de Rater and Ca de Bestiar breeds; Table S4. Summary of the results of the Mann–Whitney U test and independent median *t*-test to detect differences in the median of genetic diversity population parameters between both breeds/functionalities.
