# Supplementary material for: Does Functionality Condition the Population Structure and Genetic Diversity of Endangered Dog Breeds under Island Territorial Isolation?
Source: Animals (Basel). 2020 Oct 16;10(10):1893. doi: 10.3390/ani10101893 (PMC7602865; doi:10.3390/ani10101893)
Supplement: Supplementary file 1 [file animals-10-01893-s001.pdf]

animals

Supplementary Materials: Does Functionality Condition the Population Structure and Genetic Diversity of Endangered Dog Breeds under Island Territorial Isolation?

José Manuel Alanzor Puente [1], Águeda Laura Pons Barro [1], Manuel Rafael de la Haba Giraldo [2], Juan Vicente Delgado Bermejo [2] and Francisco Javier Navas González [2,*]

[1] Serveis de Millora Agrària i Pesquera (SEMILLA), Producció Agrària del Àrea Tècnica Àgraria, Conselleria d'Agricultura, Pesca i Alimentació, Majorca, Govern Illes Balears, 07009 Palma, Spain; jalanzor@semilla-caib.es (J.M.A.P.); apons@semilla-caib.es (Á.L.P.B.)

[2] Department of Genetics, Faculty of Veterinary Sciences, University of Córdoba, 14071 Córdoba, Spain; ge1hagim@uco.es (M.R.d.l.H.G.); juanviagr218@gmail.com (J.V.D.B.)

* Correspondence: fjng87@hotmail.com; Tel.: +34-651-679-262

Table S1. International Society of Animal Genetics (ISAG) Dog core STR panel with 21 markers showing homozygosity (Ho) and heterozygosity (He) values for each marker (ISAG, 2016).

Locus	Homozygosity (Ho)	Heterozygosity (He)
UK109	0.468	0.552
AHT126	0.526	0.662
AHT125	0.541	0.758
UK133	0.547	0.64
UK111	0.603	0.751
CXX_403	0.63	0.747
LEI007	0.646	0.746
UK118	0.654	0.807
CPH3	0.655	0.776
1_9A	0.694	0.825
UK101	0.703	0.834
CXX2137	0.753	0.893
PEZ5	0.444	0.488
PEZ20	0.456	0.492
FHC2010	0.475	0.508
PEZ1	0.515	0.566
PEZ6	0.604	0.653
PEZ12	0.639	0.649
PEZ8	0.64	0.689
PEZ13	0.663	0.684
PEZ11	0.718	0.720

ISAG Workshop: Applied Genetics of Companion Animals. In: Proceedings of the 35th International Conference on Animal Genetic, Salt Lake City, Utah, USA, 16-21 July, 2017.

Table S2. Generation intervals (years) and mean age (years) of the parents at the birth of their offspring for the four gametic routes in the Ca de Bestiar and Ca Rater dog breeds.

**Parameter / Population set**		**Gametic route**	**Male to son**	**Female to son**	**Male to daughter**	**Female to daughter**	**Total**
Ca de Bestiar	Historical (n=385)	N	16	29	15	29	89
		Mean	3.51	2.71	3.51	3.02	3.09
		Standard deviation	2.79	2.10	1.81	2.34	2.26
		Standard error of the mean	0.70	0.39	0.47	0.44	0.24
	Current (n=307)	N	9	14	9	15	47
		Mean	5.20	3.79	4.02	3.60	4.04
		Standard deviation	2.61	2.41	1.44	2.65	2.38
		Standard error of the mean	0.87	0.80	0.48	0.88	0.35
Ca Rater	Historical (n=1810)	N	59	108	55	107	329
		Mean	3.77	4.33	3.78	3.74	3.94
		Standard deviation	2.45	2.95	1.96	2.08	2.45
		Standard error of the mean	0.32	0.28	0.26	0.20	0.14
	Current (n=1468)	N	53	88	50	87	278
		Mean	3.79	4.48	3.64	3.64	3.93
		Standard deviation	2.42	3.02	1.92	1.99	2.45
		Standard error of the mean	0.33	0.42	0.26	0.27	0.15
Parameter / Population set		Mean age of the parents at the birth of their offspring	Male to son	Female to son	Male to daughter	Female to daughter	Total
Ca de Bestiar	Historical (n=385)	N	92	98	89	98	377
		Mean	4.52	3.83	4.80	3.72	4.20
		Standard deviation	2.73	2.85	3.82	1.98	2.92
		Standard error of the mean	0.28	0.29	0.41	0.20	0.15
	Current (n=307)	N	79	77	75	78	309
		Mean	5.01	4.42	5.10	4.06	4.64
		Standard deviation	2.61	2.90	4.01	1.85	2.95
		Standard error of the mean	0.29	0.33	0.45	0.21	0.17
Ca Rater	Historical (n=1810)	N	691	725	684	713	2813
		Mean	4.06	4.27	3.84	3.86	4.01
		Standard deviation	2.55	2.61	2.38	2.28	2.46
		Standard error of the mean	0.10	0.10	0.09	0.09	0.05
	Current (n=1468)	N	612	641	607	632	2492
		Mean	4.06	4.27	3.75	3.87	3.99
		Standard deviation	2.54	2.60	2.28	2.25	2.43

Standard error of the mean	0.10	0.11	0.09	0.09	0.05

Table S3. Summary of the descriptive statistics of genetic diversity population parameters for Ca de Rater and Ca de Bestiar breeds.

Breed	Parameter	F, %	ΔR, %	C, %	NRM	Maximum Generations	Complete Generations	Equivalent Generations	ΔF, %	GCI
Ca de Rater	Mean	0.01	0.02	0.01	0.00	2.35	1.04	1.50	0.01	2.98
	Std. Error of Mean	0.00	0.00	0.00	0.00	0.05	0.02	0.03	0.00	0.05
	Median	0.00	0.02	0.01	-0.01	2.00	1.00	1.50	0.00	2.29
	Mode	0.00	0.00	0.00	0.00	1.00	1.00	1.00	0.00	2.00
	Std. Deviation	0.04	0.02	0.01	0.04	2.09	0.79	1.09	0.05	1.96
	IQR	0.26	0.08	0.04	0.29	8.00	4.00	5.04	0.44	10.80
	Minimum	0.00	0.00	0.00	-0.04	0.00	0.00	0.00	0.00	1.00
	Maximum	0.26	0.08	0.04	0.25	8.00	4.00	5.04	0.44	11.80
	Sum	20.82	45.05	22.52	-1.77	4248.00	1890.00	2722.77	16.65	5385.98
	Percentile 25	0.00	0.01	0.00	-0.02	1.00	1.00	1.00	0.00	1.78
	Percentile 75	0.00	0.04	0.02	0.00	3.00	1.00	2.13	0.00	3.62
Ca de Bestiar	Mean	0.00	0.02	0.01	-0.01	1.72	0.53	0.89	0.00	1.99
	Std. Error of Mean	0.00	0.00	0.00	0.00	0.11	0.03	0.05	0.00	0.06
	Median	0.00	0.01	0.01	0.00	1.00	0.00	0.50	0.00	1.33
	Mode	0.00	0.00	0.00	0.00	0.00	0.00	0.00	0.00	1.00
	Std. Deviation	0.02	0.02	0.01	0.02	2.21	0.62	0.98	0.01	1.25
	IQR	0.13	0.06	0.03	0.14	7.00	2.00	3.72	0.05	5.94
	Minimum	0.00	0.00	0.00	-0.03	0.00	0.00	0.00	0.00	1.00
	Maximum	0.13	0.06	0.03	0.11	7.00	2.00	3.72	0.05	6.94
	Sum	1.04	6.65	3.32	-2.32	663.00	205.00	341.69	0.49	767.92
	Percentile 25	0.00	0.00	0.00	-0.01	0.00	0.00	0.00	0.00	1.00

	Percentile 75	0.00	0.03	0.01	0.00	3.00	1.00	1.75	0.00	2.91

Table S4. Summary of the results of Mann Whitney's U test and Independent Median test to detect differences in the median of genetic diversity population parameters between both breeds/functionalities.

**Items**	**F, %**	**ΔR, %**	**C, %**	**NRM**	**Maximum Generations**	**Complete Generations**	**Equivalent Generations**	**ΔF, %**	**GCI**
Total N	2195.000	2195.000	2195.000	2195.000	2195.000	2195.000	2195.000	2195.000	2195.000
Mann-Whitney U test									
Mann-Whitney U	315846.500	282642.000	282642.000	370779.000	266547.000	224355.000	242971.000	315967.500	241374.500
Wilcoxon W	390151.500	356947.000	356947.000	445084.000	340852.000	298660.000	317276.000	390272.500	315679.500
Test Statistic	315846.500	282642.000	282642.000	370779.000	266547.000	224355.000	242971.000	315967.500	241374.500
Standard Error	6452.936	11285.123	11285.123	11285.123	11100.848	10411.539	11152.646	6452.992	11159.834
Standardized Test Statistic	-5.049	-5.829	-5.829	1.981	-7.376	-11.917	-9.456	-5.030	-9.592
Asymptotic P-value (2-sided test)	0.001	0.001	0.001	0.048	0.001	0.001	0.001	0.001	0.001
Total N	2195.000	2195.000	2195.000	2195.000	2195.000	2195.000	2195.000	2195.000	2195.000
Independent Median test									
Median	0.001	0.021	0.011	-0.008	2.000	1.000	1.500	0.001	2.286
Test Statistic	25.387	29.104	29.104	16.481	10.783	61.473	27.804	25.387	17.359
Degree Of Freedom	1.000	1.000	1.000	1.000	1.000	1.000	1.000	1.000	1.000
Asymptotic P-value (2-sided test)	0.001	0.001	0.001	0.001	0.001	0.001	0.001	0.001	0.001
Yates's Continuity Correction Chi-Square	24.534	28.502	28.502	16.029	10.404	60.404	27.199	24.534	16.894
Yates's Continuity Correction Degree Of Freedom	1.000	1.000	1.000	1.000	1.000	1.000	1.000	1.000	1.000
Yates's Continuity Correction Asymptotic Sig.(2-sided test)	0.001	0.001	0.001	0.001	0.001	0.001	0.001	0.001	0.001

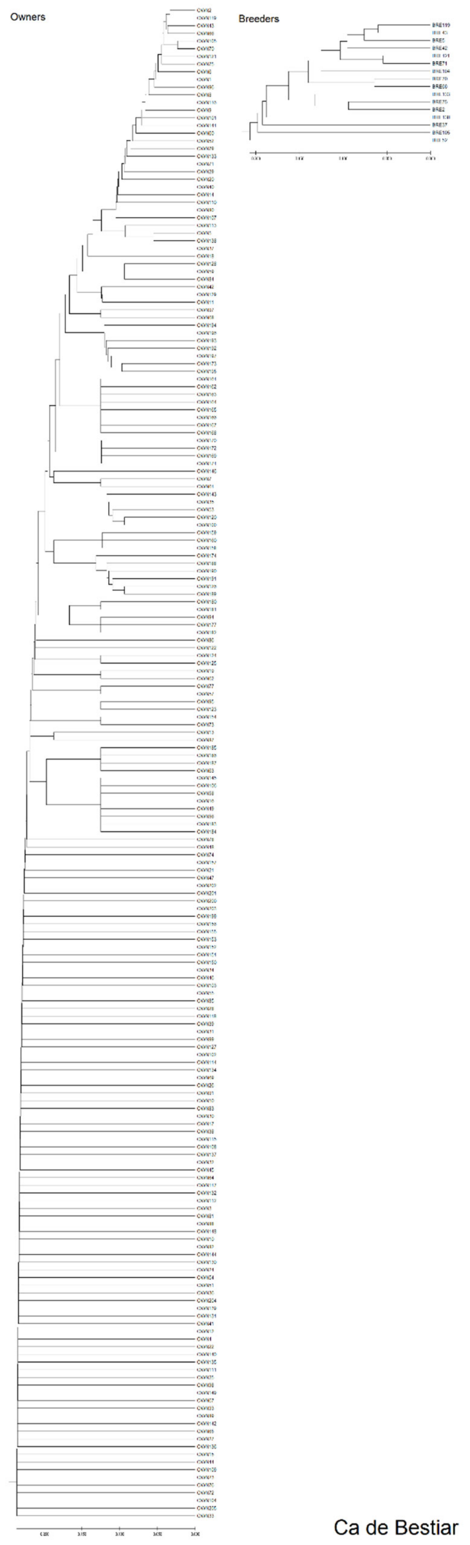

Figure S1. Dendrograms constructed from Nei's genetic distances between owners and breeders in Ca de Bestiar breed.

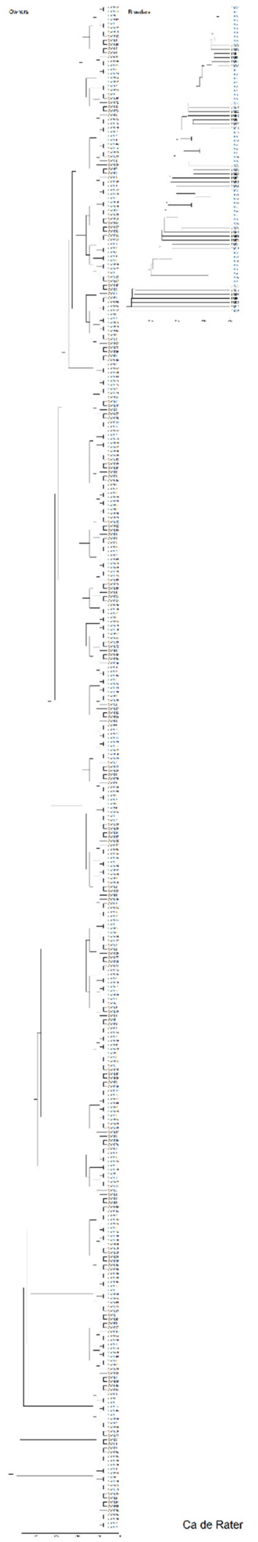

Figure S2. Dendrograms constructed from Nei's genetic distances between owners and breeders in Ca de Rater breed.
